# Role of Post-Operative Rehabilitation in TM Joint Arthritis: Functional Outcomes of Interposition Trapeziectomy vs. Prosthesis

**DOI:** 10.3390/jfmk10020198

**Published:** 2025-05-30

**Authors:** Camillo Fulchignoni, Silvia Pietramala, Leopoldo Arioli, Emanuele Gerace, Domenico De Mauro, Giulia Frittella, Elisa Di Dio, Mirko Grauso, Gianfranco Merendi, Lorenzo Rocchi

**Affiliations:** 1 Orthopaedics and Hand Surgery Unit, Fondazione Policlinico A. Gemelli IRCCS, 00168 Rome, Italy; camillo.fulchignoni2@guest.policlinicogemelli.it (C.F.); leopoldo.arioli@guest.policlinicogemelli.it (L.A.); emanuele.gerace@gmail.com (E.G.);; 2 Department of Orthopaedics, Aeging and Rheumatology Sciences, Fondazione Policlinico A. Gemelli IRCCS-Università Cattolica del Sacro Cuore, 00168 Rome, Italy; demaurodomenico@gmail.com (D.D.M.); mirko.grauso@policlinicogemelli.it (M.G.); 3 Orthopaedic Unit, Department of Public Health, Federico II University, 80131 Naples, Italy

**Keywords:** trapeziometacarpal osteoarthritis, rehabilitation, TMC prosthesis

## Abstract

**Background**: Trapeziometacarpal (TM) joint arthritis is a common condition causing pain and functional limitations, particularly in activities requiring pinch and grip strength. Surgical options such as interposition trapeziectomy and prosthetic joint replacement have demonstrated varying degrees of success. However, the role of post-operative rehabilitation in optimizing outcomes for these procedures remains underexplored. Effective rehabilitation may be critical for restoring strength, range of motion (ROM), and overall hand function; yet, no consensus exists on the best approach for either surgical modality. This study aims to evaluate the impact of post-operative rehabilitation on functional and clinical outcomes in patients undergoing interposition trapeziectomy versus prosthetic replacement for TM joint arthritis. **Methods**: A retrospective cohort study was conducted on patients treated for TM joint arthritis between November 2023 and October 2024. Patients were divided into two groups based on the surgical procedure: interposition trapeziectomy and prosthetic replacement. Patients randomly followed post-operative rehabilitation protocols, auto-assisted exercises, or no type of rehabilitation. The outcomes assessed included pain (VAS), return to work or heavy activities, post-operative complications, hand function (DASH score), and patient satisfaction at 1 and 6 months after surgery. **Results**: The prosthesis group consisted of 30 patients, while 31 patients underwent interposition trapeziectomy. Patients in both groups showed good improvements in pain, ROM, and hand function post rehabilitation. The prosthetic group demonstrated a faster recovery of strength and higher early satisfaction scores, but in the long term, the results were overlapping. No significant differences were observed in long-term functional outcomes or patient satisfaction at 6 months. In the trapeziectomy group, for patients who followed a rehabilitation program, no significant differences were found. **Conclusions**: Post-operative rehabilitation finds its place in recovery after both interposition trapeziectomy and prosthetic replacement for TM joint arthritis. While prosthetic replacement allows for quicker functional recovery, interposition trapeziectomy offers comparable long-term results with a lower complication profile. Tailored rehabilitation protocols may enhance outcomes and should be considered an integral part of TM joint arthritis management in selected patients.

## 1. Introduction

Rhizarthrosis is a degenerative condition affecting the trapeziometacarpal (TMC) joint and is characterized by progressive cartilage deterioration, chronic inflammation, and the formation of osteophytes and bone cysts involving the trapezium and first metacarpal bone [1]. The joint’s wide range of motion and inherent instability contribute to its vulnerability [2]. Female sex is a recognized risk factor, likely due to greater ligamentous laxity [3,4]. Occupations involving repetitive thumb use and heavy manual labor also increase the risk [3]. A genetic predisposition is suggested by the familial clustering of the disease [5].

The TMC joint is the second most common site of hand osteoarthritis (OA) after the distal interphalangeal (DIP) joint of the index finger. Radiographic signs appear in up to 40% of women over 80, although symptoms vary widely and may be absent in early stages [2,6]. When present, symptoms include pain, swelling, and stiffness at the thumb base, exacerbated by grip or pinch activities. Over time, functional limitation and reduced range of motion may develop [7].

Diagnosis is based on clinical evaluation and standard hand radiographs (PA, lateral, and oblique views). CT scans are reserved for complex preoperative planning [8]. The Eaton–Littler classification grades the severity based on joint space narrowing, sclerosis, osteophytes, and cysts, while Dell’s classification incorporates the degree of metacarpal base subluxation and the presence of pantrapezial arthritis [9,10].

Management aims to relieve pain, restore function, and limit disease progression. Conservative measures include activity modification, splinting, analgesics, and corticosteroid injections [11]. Surgery is indicated for advanced stages or failure of nonoperative treatment.

Surgical options include arthrodesis, denervation [12], arthroscopy [13], and primarily trapeziectomy—with or without tendon interposition—or TMC joint replacement. Trapeziectomy alone may lead to shortening and impingement, often addressed by combining it with tendon interposition or ligament reconstruction [14,15]. The most common method, Ligament Reconstruction and Tendon Interposition (LRTI), uses half of the flexor carpi radialis or abductor pollicis longus tendon to reconstruct the anterior oblique ligament [15]. A survey by the American Society for Surgery of the Hand found that trapeziectomy with LRTI remains the preferred option, despite no proven superiority over simple trapeziectomy [16,17].

Total TMC joint arthroplasty was introduced in the 1970s [18], with subsequent prosthetic designs emulating a small hip joint [19]. After two decades of follow-up, it has become a well-established procedure, particularly for Eaton stages II and III [20,21]. Currently, there is no established standard for the surgical treatment of basal thumb arthritis. Recent systematic reviews indicate a faster recovery in the TMC implant groups but no significant differences in long-term outcomes [7].

A significant gap in knowledge exists regarding post-operative rehabilitation protocols following trapeziectomy. While various authors suggest different approaches, there is no consensus or standardized guideline to support clinical decision making. Typically, a thumb cast or splint is applied for 2 to 3 weeks, followed by a thermoplastic splint for an additional 4 weeks. However, the decision to refer patients to hand therapy or to instruct them in self-assisted exercises remains largely dependent on individual surgeon preference. This lack of standardization highlights the need for further research to define the role and timing of post-operative rehabilitation, which many authors nonetheless consider an integral part of treatment [22].

To our knowledge, no prior study has directly compared the functional outcomes of patients undergoing trapeziectomy and prosthetic replacement in relation to post-operative hand therapy. Given the comparable long-term results observed with these two techniques, we aimed to investigate whether post-operative physiotherapy can influence these outcomes in terms of functional recovery, pain, and return to work.

## 2. Materials and Methods

### 2.1. Study Design

A retrospective cohort study was conducted on patients treated for trapeziometacarpal (TM) joint arthritis between November 2023 and October 2024. Patients were identified through surgical records archived at our institution (Fondazione Policlinico Universitario A. Gemelli IRCCS) using operative registries. They were subsequently contacted by telephone to obtain consent for participation in the study. The study was conducted in accordance with the ethical standards of the 1964 Declaration of Helsinki and its later amendments. An anonymized retrospective dataset was used containing no personal identifying information.

### 2.2. Participants

Patients were included in this study if they met the following criteria: (1) a confirmed clinical and radiographic diagnosis of basal thumb arthritis; (2) a clear indication for surgical treatment as determined by a specialist; and (3) a minimum post-operative follow-up period of at least six months. Exclusion criteria were (1) the presence of a concomitant rheumatic disease; (2) a history of post-traumatic arthritis; (3) neurological impairment affecting hand function; or (4) loss to follow-up.

Patients diagnosed with basal thumb arthritis and referred for surgical treatment were included in this study. Each patient was evaluated through a pre-operative radiography, including anteroposterior and lateral views. The decision to perform interposition trapeziectomy rather than joint replacement was made based on a combination of radiological and clinical factors.

Patients with Eaton–Littler stage I arthritis were considered for surgical intervention only in cases of extremely symptomatic disease that had proven refractory to conservative treatment of at least 6 months.

Patients with Eaton–Littler stage IV arthritis were treated with interposition trapeziectomy.

For patients with stage II and III arthritis, the decision-making process involved considering their functional expectations (such as occupation and age) and radiographic findings, including the adequacy of bone stock, trapezium height, and the degree of first metacarpal base subluxation.

### 2.3. Surgical Technique

In both surgical approaches, the procedure was performed under locoregional anesthesia with a tourniquet applied to the proximal upper arm. All surgeries were carried out by six experienced surgeons from the same surgical team.

#### 2.3.1. Trapeziectomy with Ligament Reconstruction and Tendon Interposition (LRTI)

A longitudinal 4 cm incision was made along the radial border of the first metacarpal. Precautions were taken to avoid damaging the branches of the radial superficial nerve. A capsule flap was elevated, and an arthrotomy was performed to access the joint. The trapezium was then exposed and subsequently excised through the capsular opening. In our institution, we employ the tendon plasty technique described by Ceruso [23], which involves using the abductor pollicis longus (APL) tendon passed around the flexor carpi radialis (FCR) tendon. The APL tendon was then sutured to the previously prepared capsule flap, ensuring that no excessive tension was applied. The plasty was completed by suturing the capsule to itself, and the skin was closed.

#### 2.3.2. Trapeziometacarpal Joint Replacement

A longitudinal lateral incision, approximately 3 cm in length and centered over the trapeziometacarpal joint, was made. A capsular flap was created to expose the base of the metacarpal, along with the APL, and the trapezium was exposed.

In our institution, the MAIA™ Lepine prosthesis is routinely used. A 5 mm resection of the base of the metacarpal was performed using the standard cutting guide, and any osteophytes were removed. An awl was then used to locate the medullary canal, and progressively larger rasps were used until the appropriate canal size was achieved. The trial stem was then inserted.

A retractor was used to enhance the visualization of the trapezium, and a Kirschner wire was inserted into its center as a guide. The trapezium was subsequently prepared using reamers of increasing sizes. The definitive prosthetic components were implanted, and joint stability was assessed. The capsule and skin were then closed. A final X-Ray was taken to verify correct implant placement.

### 2.4. Post-Operative Management

Following trapeziectomy and LRTI, a thumb spica cast was applied for 3 to 4 weeks, allowing for the free movement of the long finger and the interphalangeal joint of the thumb.

In the joint replacement group, a thumb spica cast or a soft bandage, depending on the surgeon’s preference, was indicated for a period of only 2 weeks.

Patients were assigned to the specific rehabilitation group based on their agreement to participate in the prescribed rehabilitation program after cast removal.

In the no rehabilitation group, patients received instructions on how to perform self-assisted exercises at home. These exercises included full abduction and extension of the thumb, progressive adduction, and opposition.

Patients were allowed to start the exercise program immediately at the cast/bandage removal: after 3 to 4 weeks for the trapeziectomy group and after 2 weeks for the joint replacement group. Patients were instructed to repeat the exercises 3 to 4 times a week for 15 min per session.

Lifting heavy weights and engaging in strenuous manual activities were prohibited for a minimum of 60 days after cast removal. No splint was recommended. Standard analgesic medication was prescribed for pain management.

In the rehabilitation group, a specialized hand therapist provided care to patients starting at week 4 post-operatively. Active and passive range of motion exercises were initiated, including palmar and radial abduction, wrist flexion and extension, and thumb circumduction, flexion, and extension. Scar massage was also incorporated into the therapy. During this phase, a dynamic spica splint with CMC support was recommended by the therapist for use except during demonstrations and exercise sessions. Strengthening exercises were initiated at week 8, with the goal of achieving full strength recovery by week 12. If deemed beneficial by both the surgeon and therapist, additional physical therapy modalities (e.g., ultrasound) were added to the treatment plan.

### 2.5. Outcome Measures

Patients were categorized into two groups based on the surgical procedure performed: interposition trapeziectomy and prosthetic replacement.

For each patient, demographic data were collected, including their type of occupation (manual vs. office job). The Eaton–Littler stage was assigned to each patient based on pre-operative radiographs. The duration of immobilization was determined from the post-operative follow-up visits and confirmed through communication with the patient.

The follow-up duration was calculated from the date of surgery to the date of the telephonic evaluation. However, clinical outcomes were assessed retrospectively at 6 months post-operatively by asking patients to recall their average functional status at that time. The outcomes assessed included pain (using the visual analog scale—VAS), return to work or heavy activities, the occurrence of post-operative complications, hand function (using the disabilities of the arm, shoulder, and hand—DASH score), and patient satisfaction at 1 and 6 months after surgery.

For the rehabilitation group, the number of therapy sessions attended and the type of therapy received were also recorded.

### 2.6. Statistical Analysis

All statistical analyses were performed using SPSS version 29.0. Descriptive statistics, including means, standard deviations, and standard errors, were calculated for continuous variables such as immobilization time, time to return to work, and QuickDASH scores for both intervention groups (prosthesis vs. trapeziectomy). Associations between the type of surgical intervention and categorical variables (e.g., sex, dominant side, occupation) were evaluated using chi-square tests or Fisher’s exact test as appropriate.

Linear regression models were employed to assess the effect of individual predictors (type of intervention, gender, occupational status, and physiotherapy) on the 2-month QuickDASH outcome. A multiple linear regression model was also performed to evaluate the combined influence of these factors. A *p*-value threshold of 0.05 was used to determine statistical significance, corresponding to a 95% confidence level.

## 3. Results

### 3.1. Participant Characteristics

A total of 61 patients were included in this study. The trapeziectomy group consisted of 31 patients, while joint replacement was performed on 30 patients. The cohort comprised 50 female and 11 male participants. The mean age of the patients was 64.95 years (range: 51–80 years). The dominant hand was affected in 31 patients (50.8%, not 38.75%). There were no significant differences in the operated side between the replacement and trapeziectomy groups: the right hand was operated on 16 times in each group, while the left hand was operated on 15 times in the replacement group and 14 times in the trapeziectomy group. Among the patients, 26 were active workers, of whom 13 were manual laborers. The mean follow-up period, calculated as the time between surgery and the phone call, was 9.68 months. A cast or splint was continuously worn for an average of 17.75 days, with a minimum of 15 days and a maximum of 30 days. Demographic data are showed in Table 1.

Patients referred to a hand therapist numbered 28, with an average of 14.4 sessions per patient. The type of rehabilitation was determined by the therapist and included manual therapy alone for 14 patients, while 10 patients received both manual and physical therapy. Only one patient was treated with physical therapy exclusively.

### 3.2. Clinical Outcomes by Group

The average time to return to work was 39.57 days, while the resumption of heavy manual activity occurred after an average of 80.95 days. The final Kapandji score at 6 months was 9.55. The average VAS score at 1 month was 4.16, at 3 months, it was 2.3, and the pain recorded at 6 months averaged 0.73. The average QuickDASH score at one month was 14.01, decreasing to 6.3 at six months. These statistics are reported in Table 2.

**Figure 1:**
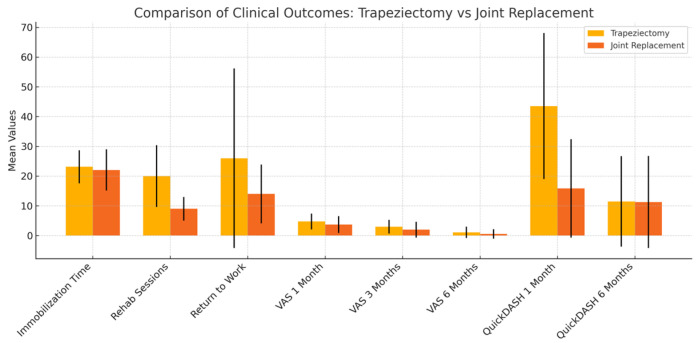
Diagram showing a comparison of outcomes of different treatments.

Data reported in Table 1 are also shown in Figure 1.

### 3.3. Role of Rehabilitation

Statistical analysis was performed by dividing patients according to rehabilitation program. In the following table, data have been determined without taking into account the type of surgery. The sole rehabilitation did not improve any of the functional outcomes evaluated except for VAS score at 3 and 6 months. Data on outcomes stratified by rehabilitation, regardless of the type of surgical procedure, are presented in Table 3.

Additional subgroups were analyzed according to assigned rehabilitation program. In the joint replacement group, no differences were noted. Patients who completed rehabilitation sessions returned to work after 31.84 days, which was slightly higher compared to the non-rehabilitation group (26,43), but this was not statistically significant. The only significant data were quickDASH at 6 months. Functional outcomes for patients who underwent joint replacement are reported in Table 4, stratified according to whether or not they received physiotherapy.

The same results were observed in the trapeziectomy group. The only significant result was the VAS at 1 month. Functional outcomes for patients who underwent trapeziectomy are reported in Table 5, stratified according to whether or not they received physiotherapy.

## 4. Discussion

### 4.1. Summary of Main Findings

Our main findings suggest that joint replacement generally leads to better short-term functional outcomes compared to trapeziectomy. However, these improved outcomes do not appear to be significantly influenced by post-operative physiotherapy. While rehabilitation remains a valuable tool—particularly in selected patients—it does not seem to play a decisive role in determining functional recovery in the broader patient population.

### 4.2. Comparison with the Literature

With an open debate regarding the best surgical treatment for thumb basal osteoarthritis, trapeziectomy with or without ligament interposition and joint replacement remain the best and most used options.

In our institution, the decision to perform one surgery over another relies on the patient’s demands and radiographic characteristics, with an overall balance in terms of numbers of surgeries. This aspect makes our data uniform, giving a realistic overview on the matter.

The two techniques have already been compared in the literature, facing different aspects especially in terms of functional outcomes and post-operative recovery.

In a recent prospective study of 147 patients, Guzzini et al. [24] reported significant improvement in both trapeziectomy and joint replacement groups, but patients treated with a prosthesis showed faster improvement in terms of strength and range of motion. QuickDASH was statistically significant only in the short term, while it was almost the same at 12 and 24 months.

Similarly, and Piccirilli et al. [25] reported better mid-term results for joint replacement when assessing the quickDASH and Kapandji scores, whereas VAS scores were registered as low in both groups.

In a 3-year non-randomized study, Falkner et al. [26] found high patient satisfaction in mid-term follow-up but superior pinch strength and shorter time of recovery for dual mobility TMC joint arthroplasty.

Our study confirmed these data in terms of time to return to work (*p*-value = 0.021) and quickDASH at 1 month (*p*-value < 0.001).

Among the various studies and reviews regarding this matter, none focused specifically on the role of rehabilitation in the different techniques. It has been pointed out that short-term satisfaction is higher in joint replacement, but rehabilitation could influence the result.

Traditionally, post-operative protocols consisted of immobilization in a cast for 4 to 6 weeks following surgery, followed by limited range of motion exercises while using a removable splint for an additional 3 to 4 weeks [27].

Overall, the standard includes up to 6 months of occupational therapy and return to heavy manual activities after 3 to 4 months [28].

Our results show that adherence to a rehabilitation program does not influence clinical outcomes in the joint replacement group nor in the trapeziectomy group (*p*-value > 0.05 in both groups).

Looking at the overall data, patients from the trapeziectomy group needed more rehabilitation sessions compared to joint replacement. These data are coherent with the poorer short-term patient satisfaction after trapeziectomy.

It must be pointed out that different hand therapists treated our patients and no standardized protocol was assigned to the two groups. Despite being a limitation of our study, this matter reflects what has already been stated in the literature. Wolff et al. [29], in fact, conclude that there is too much variation in the literature in order to formulate general recommendations on post-operative immobilization and exercises.

In order to make recommendations regarding length and type of post-operative immobilization, further research should be carried out.

No consensus has been reached regarding physical therapy, such as ultrasound or laser. In our series, 10 patients were treated with physical therapy in addition to standard manual therapy, while only 1 patient was treated with physical therapy alone. We did not find any evidence in the literature regarding the rationale of using physical therapy in the post-operative protocol for basal thumb osteoarthritis surgery despite the type of intervention.

### 4.3. Limitations of the Study

This study has several limitations. First, the sample size was relatively small, although the two treatment groups were comparable in terms of patient numbers. Second, due to the retrospective design, functional outcomes related to pain and disability at 3 and 6 months were collected via telephone interviews, which may introduce recall bias or limit the precision of the assessments. Third, no standardized rehabilitation protocol was implemented, which may have introduced variability in the type, duration, and intensity of physiotherapy received. Finally, patients could not be randomized to receive rehabilitation due to socioeconomic factors, including limited access to specialized physiotherapy services, high out-of-pocket costs, and geographical barriers for those living far from the treatment center. These factors may have influenced the rehabilitation pathway independently of clinical indications.

### 4.4. Clinical Implications

The findings of this study suggest that, while joint replacement may offer superior short-term functional outcomes compared to trapeziectomy, the addition of post-operative rehabilitation does not appear to significantly influence these results in the general patient population. This highlights the importance of tailoring physiotherapy to selected cases rather than applying it routinely. In clinical practice, this may help optimize resource allocation and reduce unnecessary costs, especially in healthcare systems where access to specialized rehabilitation is limited by socioeconomic or geographic factors.

### 4.5. Future Research Directions

Further research is needed to validate our findings in larger prospective cohorts. Randomized controlled trials comparing standardized rehabilitation protocols to no rehabilitation could help clarify the real impact of physiotherapy on functional outcomes after both trapeziectomy and joint replacement. It would also be valuable to explore which subgroups of patients—based on factors such as age, baseline function, or comorbidities—may benefit the most from targeted rehabilitation. Finally, future studies should consider the cost-effectiveness of personalized rehabilitation strategies, particularly in healthcare systems where access to specialized physiotherapy is limited.

## 5. Conclusions

Rehabilitation is often considered essential in hand surgery, but there is no established consensus on its role following surgery for basal thumb osteoarthritis. Our findings suggest that, while joint replacement may offer faster functional recovery than trapeziectomy, the long-term outcomes are comparable. Post-operative rehabilitation can be beneficial, but it does not significantly influence overall functional results and should be reserved for selected patients with greater functional needs or limited ability to perform self-directed exercises.

## Figures and Tables

**Table 1:** Demographic data.

Type of surgery	Trapeziectomy	31
	Joint replacement	30
Gender	Male	11
	Female	50
Mean Age	64.95 years old	Range 51–80
Affected hand	Right	32
	Left	29
Eaton–Littler stage	I	0
	II	22
	III	35
	IV	4
Follow-up	9.68 months	Range 6–35

**Table 2:** Trapeziectomy vs. joint replacement statistics.

	Trapeziectomy	Joint Replacement	*p*-Value
Rehabilitation	17	11	
Time of immobilization (m, SD)	23.12 ± 5.53	22.06 ± 6.95	0.153
N° rehabilitation sessions (m, SD)	20 ± 10.38	9 ± 3.97	**0.015**
Return to work (days) (m, SD)	26 ± 30.18	14 ± 9.89	**0.021**
VAS 1 month (m, SD)	4.73 ± 2.65	3.71 ± 2.84	0.195
VAS 3 months (m, SD)	3.00 ± 2.29	1.97 ± 2.67	0.120
VAS 6 months (m, SD)	1.07 ± 1.92	0.58 ± 1.57	0.309
quickDASH 1 month (m, SD)	43.53 ± 24.54	15.87 ± 16.53	**<0.001**
quickDASH 6 months (m, SD)	11.48 ± 15.19	11.29 ± 15.46	0.967

m = mean, SD = standard deviation, VAS = visual analog scale, DASH = disability of the arm, shoulder, and hand.

**Table 3:** Statistical data on rehabilitation vs. no rehabilitation subgroups.

	Rehab	No Rehab	*p*-Value
Return to work (days) (m, SD)	41.21 ± 33.9	33.57 ± 27.4	0.14
VAS 1 month (m, SD)	4.76 ± 2.87	3.75 ± 2.65	0.08
VAS 3 months (m, SD)	3.16 ± 2.54	1.86 ± 2.36	**0.02**
VAS 6 months (m, SD)	1.56 ± 2.45	0.29 ± 1.14	**0.03**
quickDASH 1 month (m, SD)	33.85 ± 27.50	25.54 ± 22.48	0.10
quickDASH 6 months (m, SD)	13.10 ± 17.80	6.95 ± 14.08	0.06

m = mean, SD = standard deviation, VAS = visual analog scale, DASH = disability of the arm, shoulder, and hand.

**Table 4:** Statistical data on joint replacement subgroups.

	Rehab	No Rehab	*p*-Value
Return to work (days) (m, SD)	31.84 ± 10.68	26.43 ± 6.26	0.223
VAS 1 month (m, SD)	3.39 ± 2.95	4.63 ± 2.46	0.298
VAS 3 months (m, SD)	1.67 ± 2.61	2.75 ± 2.83	0.340
VAS 6 months (m, SD)	0.25 ± 0.78	1.67 ± 2.85	0.052
quickDASH 1 month (m, SD)	16.01 ± 17.35	15.31 ± 15.31	0.919
quickDASH 6 months (m, SD)	6.66 ± 10.54	25.00 ± 20.58	**0.016**

m = mean, SD = standard deviation, VAS = visual analog scale, DASH = disability of the arm, shoulder, and hand.

**Table 5:** Statistical data on trapeziectomy subgroups.

	Rehab	No Rehab	*p*-Value
Return to work (days) (m, SD)	51.63 ± 33.94	43.33 ± 15.34	0.356
VAS 1 month (m, SD)	5.4 ± 2.8	3.61 ± 2.25	**0.041**
VAS 3 months (m, SD)	3.4 ± 2.50	2.41 ± 2.19	0.147
VAS 6 months (m, SD)	1.42 ± 2.45	0.58 ± 1.44	0.139
quickDASH 1 month (m, SD)	46.62 ± 26.39	40.20 ± 22.00	0.247
quickDASH 6 months (m, SD)	13.55 ± 17.08	9.7 ± 14.08	0.278

m = mean, SD = standard deviation, VAS = visual analog scale, DASH = disability of the arm, shoulder, and hand.

## Data Availability

All relevant data are published in the manuscript. Any additional data, including detailed statistical analyses, are available upon request.

## Abbreviations

The following abbreviations are used in this manuscript:

CMC	Carpo-metacarpal
OA	Osteoarthritis
DIP	Distal interphalangeal
TMC	Trapezio-metacarpal
AOL	Anterior oblique ligament
LRTI	Ligament reconstruction tendon interposition
APL	Abductor pollicis longus
FCR	Flexor carpi radialis
VAS	Visual analog scale
DASH	Disability of the arm, shoulder, and hand
